# Human infectious diseases in the genomics era: where do we go from here?

**DOI:** 10.1186/s13059-014-0529-5

**Published:** 2014-11-22

**Authors:** Ripudaman K Bains

**Affiliations:** Genome Biology, BioMed Central, 236 Gray’s Inn Road, London, WC1X 8HB UK

## Abstract

Ripudaman K Bains is the editor of the *Genome Biology* special issue content on the ‘genomics of infectious diseases’, and introduces the collection in this editorial.

## Editorial

Over the past few years there has been what can only be described as an explosion in the field of pathogen genomics. This has been predominantly facilitated by the availability of quicker and cheaper sequencing technologies, but also, and not insignificantly, by substantial political pressure and increased funding from numerous governmental and philanthropic organisations. Despite this, the devastating global burden of infectious diseases is far from being alleviated; the recent outbreak of Ebola virus serves as a reminder of how infectious diseases continue to disproportionately affect the truly destitute in economically developing regions. However, the landscape of infectious diseases is changing, as demonstrated by the recent re-emergence of Dengue fever in Japan, for the first time in 70 years [1]. This has reignited concerns about shifting transmission dynamics of communicable diseases, including how climate change is likely to affect their global spread, and it has reiterated the need for political action to meet challenges in the near future.

It is against this backdrop that *Genome Biology* and *Genome Medicine* publish this collaborative and particularly timely issue on the ‘genomics of infectious diseases’. In this endeavour, we are indebted to our Guest Editors, Professor George Weinstock and Professor Sharon Peacock, for their invaluable advice and guidance. We are also extremely grateful to all authors who submitted manuscripts, and to the many reviewers who provided feedback and advice.

## The rise of genomic approaches to understanding infectious diseases

Genomic approaches have not always been available for infectious disease research, or for guiding prevention programmes. Some of the most ambitious and successful eradication programmes of the 20th century utilised vaccine distribution strategies or environmental approaches. These included the Global Polio Eradication Initiative, which began in the 1950s and involved a global immunisation programme, and the Guinea Worm Eradication Program, which focused on improving sanitation and access to clean and safe water supplies.

The potential for genomic studies to transform our understanding of pathogens was initially realised with the completion of the first pathogen sequencing project: that of *Haemophilus influenzae* in 1995, after decades of work [2]. *Encephalitozoon cuniculi* was the next pathogen to have its genome completely sequenced in 2001 [3], followed by *Plasmodium falciparum* in 2002 [4]. The significance of these pioneering genomic studies should not be underestimated; since their completion, genome approaches have revolutionised the study of pathogens. For example, for some infectious diseases, genomics-based studies have elucidated how and why drug resistance emerges by identifying genomic signatures. They have also enabled researchers to retrospectively analyse where and why the outbreak of a particular disease occurred; examples of such an approach include studies of Legionnaires’ disease [5], severe acute respiratory syndrome [6], bird influenza [7], and global epidemics - including the current Ebola outbreak that is ravaging West Africa [8]. Sequencing technologies themselves also have incredible potential for diagnosing previously uncharacterised pathogens, as shown in the *Genome Biology* article on a rare brain tapeworm [9]. Genomics therefore has an important and multifaceted role in the future control of infectious diseases.

The increased number of genomic studies of infectious diseases has not been without controversy. One of the most highly publicised examples being Ron Fouchier’s 2012 *Science* publication, which showed how influenza viruses could acquire mutations and become pathogenic, a feat for which he was named one of Time magazine’s 100 most influential people of 2012 [10]. After a lengthy court battle, a Dutch district court in Haarlem ruled that Fouchier had not followed European Union restrictions on preventing the proliferation of weapons of mass destruction and so-called dual-use technology that could be used for good or evil [11]. The court’s decision led to heated debates about the importance of academic freedom, and concerns about the future of infectious disease genomics research.

## Where do we go from here?

Despite the potential setback of the Fouchier ruling, genomic approaches continue to be utilised to address a wide range of questions in pathogen research, which is evidenced by topics presented in this special issue. The use of chemotherapy has been a mainstay of disease control programmes since the 1950s, and the emergence of drug resistance remains a global health concern. In one of our featured reviews, Elizabeth Winzeler and Micah Manary discuss the growing problem of artemisinin resistance in malaria-endemic regions [12], and how genomic studies have identified the signatures of resistance.

In another review [13], Flaminia Catteruccia and colleagues discuss different approaches for engineering mosquito populations. This review is particularly timely given the 2012 ruling by the Brazilian government to allow a longitudinal field trial of genetically modified mosquitoes for the control of Dengue fever [14]. This decision was not without opposition, perhaps most notably from environmental groups. In light of the recent re-emergence of infectious diseases in many areas of the world, this controversial method of vector control is likely to become more commonplace.

In the midst of the excitement about high-throughput approaches, it is important to remember that such methods are not always affordable or practical in endemic regions. As shown in the *Genome Biology* paper by Pardis Sabeti and colleagues [15], the optimisation of existing, accurate and cheap technologies is a suitable option for the rapid diagnosis of Ebola and Lassa viruses. In the drive towards ever more sophisticated technologies, this work is a humbling reminder that they are not always an option in regions in which the problems are most pressing.

## Empowering genomics research at regional and international levels

The ongoing Ebola crisis has highlighted the need for co-ordinated international responses to tackle disease outbreaks. This is only possible through collaborative research, and in recent months a number of institutions have been established to address this need at both regional and international levels.

In a Comment article from Christian Happi and colleagues [16], the authors address the imbalance between genomics research capabilities within and outside Africa, a continent with a significant proportion of the global burden of infectious diseases. The establishment of the African Center of Excellence for Genomics of Infectious Diseases empowers African genomicists to take the lead in developing solutions for their control. This institute is an indication of how economically developing regions are likely to increasingly take the lead in collaborative research projects to help address both regional and global problems.

## Genome Biology and infectious disease genomics

*Genome Biology* received over 100 manuscripts for consideration in this special issue. The wealth of topics covered in the submissions demonstrates not only how far the field has come but also how exciting and broad the research continues to be. It is clear that genomics has a vital role in research into the biology of infectious diseases, as well as in finding innovative solutions to their control. The journal will continue to publish novel and important research within this area following this special issue.

## Concluding remarks

We hope that you find this collection of articles interesting, informative and useful. However, it is with regret that we must also mention that an author of one of the published manuscripts sadly passed away in Sierra Leone after losing his fight against Ebola, after working to collect strains of the virus. We hope that in addition to showcasing cutting-edge research into human pathogen genomics, this special issue also reminds our readers of the devastation that these diseases continue to cause, and the risks to those who work on the frontline to defeat them.
